# Revision of the legume-feeding leaf beetle genus *Madurasia* Jacoby, including a new species description (Coleoptera, Chrysomelidae, Galerucinae, Galerucini)

**DOI:** 10.3897/zookeys.597.7520

**Published:** 2016-06-09

**Authors:** Kaniyarikkal Divakaran Prathapan

**Affiliations:** 1 Department of Entomology, Kerala Agricultural University, Vellayani P.O., Trivandrum–695 522, Kerala, India

**Keywords:** Asia, Africa, biology, pest, pulses, taxonomy

## Abstract

*Madurasia* Jacoby is revised and *Madurasia andamanica* **sp. n.**, endemic to the Andaman Islands in the Indian Ocean, is described and illustrated. *Madurasia obscurella* Jacoby, **syn. n.**, is a new junior synonym of *Madurasia undulatovittata* (Motschulsky), **comb. n.** A lectotype is designated for *Madurasia obscurella*. Literature on the biology and management of *Madurasia undulatovittata* is reviewed.

## Introduction

The monotypic galerucine genus *Madurasia* was described by Jacoby (1886) for a new species, *Madurasia obscurella*, from southern India. Aslam (1972) synonymized *Neorudolphia bedfordi* Laboissière, 1926, the only species in this monotypic genus from Sudan, with *Madurasia obscurella* Jacoby. Examination of the type of *Monolepta undulatovittata* (Motschulsky 1866) (originally described in *Teinodactyla* Chevrolat = *Longitarsus* Latreille) from Sri Lanka has shown that *Madurasia obscurella* is a junior synonym of Motschulsky’s species. The genus is here revised and a new species is described from the Andaman Islands in the Indian Ocean. Information on the biology, pest status and management of *Madurasia undulatovittata* comb. n., which is a significant pest of various legume crops in south-east Asia and Africa, is reviewed.

## Materials and methods

Dissecting techniques and descriptive terminology follow Konstantinov (1998). Label data for holotypes, lectotypes, and paralectotypes has been recorded verbatim, with lines on the same label separated by “/” and labels separated by “;”. Material examined is from the following collections:

BMNH
Natural History Museum, London



INPC
National Pusa Collection, Indian Agricultural Research Institute, New Delhi 


JBC
 Personal collection of Jan Bezděk, Czech Republic 


KAU
 Travancore Insect Collection, Kerala Agricultural University, Vellayani 


NBAIR
 National Bureau of Agricultural Insect Resources, Bangalore 


UASB
University of Agricultural Sciences, Bengaluru 


USNM
National Museum of Natural History, Smithsonian Institution, Washington D.C. 


ZMUH
Zoologisches Institut und Zoologisches Museum, Universität von Hamburg, Hamburg, Germany 


ZMUM
Zoological Museum, Moscow State University, Moscow 


Determination of the gender of the undissected specimens is provisional as sexually dimorphic characteristics are often not clearly discernible externally.

## Systematics

### Madurasia

Taxon classificationAnimaliaColeopteraChrysomelidae

Jacoby, 1886

Madurasia Jacoby, 1886: 280 (Type species: Madurasia obscurella Jacoby, 1886, southern India, by monotypy)–Maulik 1936: 72–Wilcox 1973: 435–Seeno and Wilcox 1982: 107–Jolivet and Hawkeswood 1995: 101 (host plants)–Medvedev and Sprecher–Uebersax 2005: 316 (key)–Beenen 2010: 481.
Neorudolphia Laboissière, 1926: 190 (Type species: Neorudolphia bedfordi Laboissière, 1926, Sudan, by monotypy)–Wilcox 1973: 435–Aslam 1972: 500 (= MadurasiaJacoby 1886: 280).

#### Description.

**Figures 1–7.**
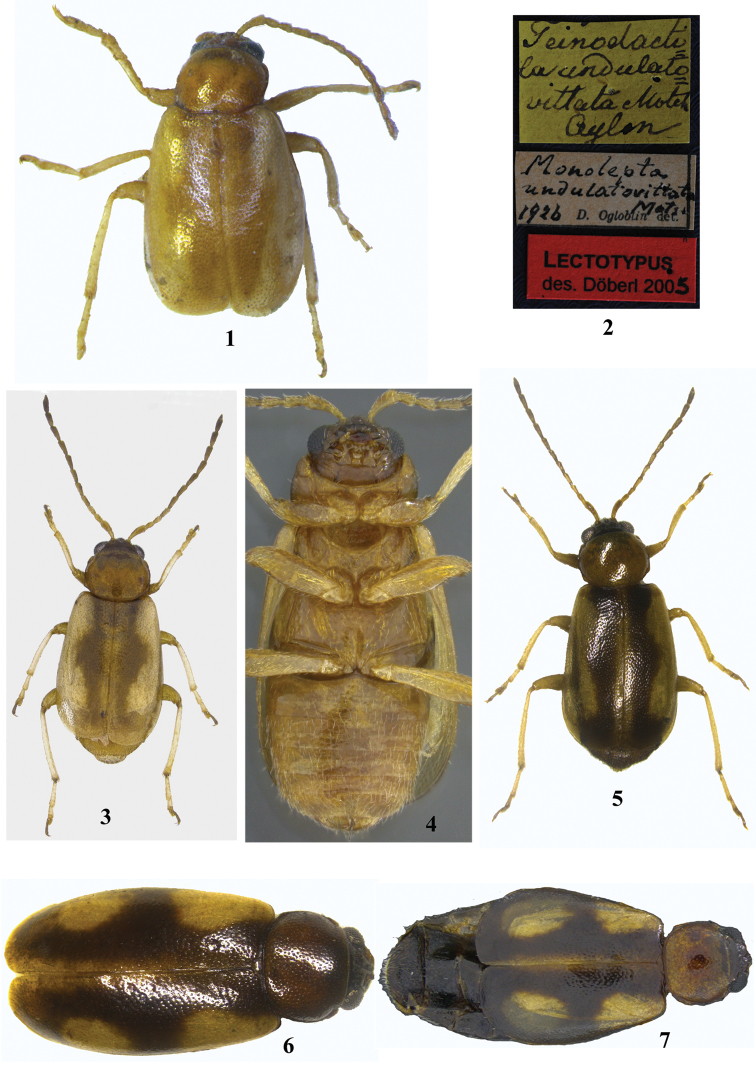
*Madurasia undulatovittata*. **1** Lectotype (specimen on card, photograph edited) **2** labels on lectotype **3** and **5–7** dorsal view, color variation **4** ventral view.

Body: length 2.0–3.0 mm; 1.8–2.3 times longer than wide. Moderately small, oblong, flattened in lateral view, length 3.1–3.4 times height. General color straw brown to dark brown with a characteristic, more or less distinct, dark, broad longitudinal stripe on each elytron (Figs 1, 3, 5–7, 21); mesal margin of stripe nearly straight; each stripe nearer to suture than to lateral margin of elytron; stripe narrowing laterally posteriorly of humerus and in distal 2/3 of elytron.

**Figures 8–20.**
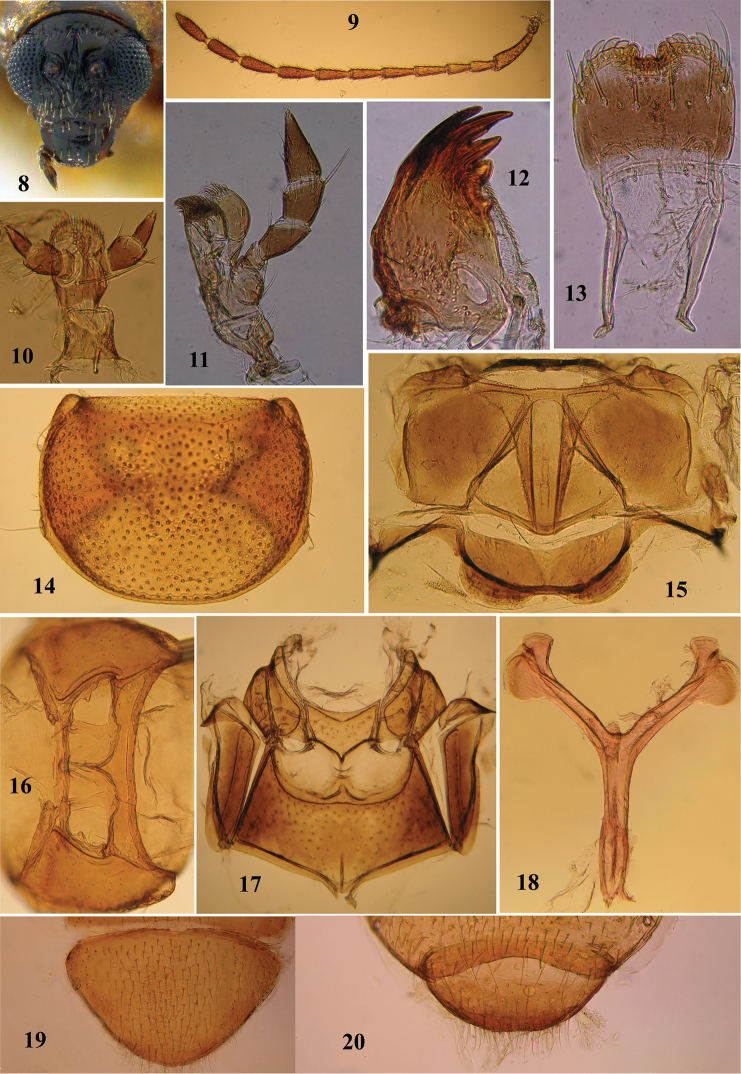
*Madurasia undulatovittata*. **8** head, frontal view **9** antenna **10** labium **11** maxilla **12** mandible **13** labrum **14** pronotum **15** meso– and metanotum **16** prosternum **17** meso– and metasternum and pleurites **18** metendosternite **19** apical visible tergite, female **20** apical visible tergite, male (all specimens, except head, have been macerated).

Head (Fig. 8) hypognathous with frontal view slightly longer than wide. In lateral view anterior margin forms a moderately convex line with a notch where vertex meets antennal calli and a second notch at anterior end of frontal ridge. Supraorbital pore represented by a large setaceous pore adjacent to orbital sulcus near eye. Seta in supraorbital pore upcurved. Vertex shiny, indistinctly wrinkled, nearly impunctate. Antennal calli trapezoidal, longer than wide, moderately convex, raised above adjacent border of vertex, separated from each other by a deep midfrontal sulcus; anterior ends acutely triangular, enter into interantennal space, reaching well below midlevel of antennal socket. Orbital sulcus short, deep, represented by supraorbital pore and adjacent area. Supracallinal sulcus represented by punctures arranged in an irregular transverse row, each puncture in supracallinal row bearing a short down-curved seta. Midcranial suture absent. Supraorbital sulcus less distinct than midfrontal sulcus. Suprafrontal and supraantennal sulci well defined. Subgenal suture distinct. Transverse diameter of eye 5.2–8.8 times distance between eye and antennal socket, 2.9–4.4 times distance between antennal sockets, 1.7–1.9 times width of antennal socket, 0.6–0.7 times distance between eyes. Eyes lateral, medium sized, convex, inner margins indistinctly concave, and ventrally divergent. Frontal ridge narrowest between antennal sockets, joins anterofrontal ridge anteriorly. Anterofrontal ridge transverse, gently curved. Frontal ridge together with antero-frontal ridge forms T–shaped ridge. Anterofrontal ridge lower than frontal ridge. Frontolateral area coarsely punctate, each puncture bearing a long seta. Frontoclypeal suture with a row of eight setae. Clypeus narrow. Visible part of labrum much wider than long, with a transverse row of eight pores; all eight pores in *Madurasia andamanica* sp. n. with a well-developed seta; while only six pores, excluding third pore from either end, with seta in *Madurasia undulatovittata*. Labrum (Fig. 13) with anterior margin incised medially; about seven sensillae on either side of incision, arranged along anterior margin of labrum’s inner surface; tormae longer than width of labrum. Mandible (Fig. 12) palmate with six sharp denticles. Maxilla (Fig. 11) with four palpomeres: first shortest, second and third subequal, both longer than first, but shorter than apical palpomere, apical longest; lacinia wider than galea. Labial palpi (Fig. 10) with three palpomeres, basal two wider than long, middle widest, apical palpomere longer than wide and longest of three. Antenna (Fig. 9) reaches more or less middle of elytron. First antennomere longest, club shaped; second smallest; third a little longer than second; fourth distinctly longer than third; 4–10 subequal in length; eleventh longer than all except first antennomere; five to six distal antennomeres wider than preceding three or four (Fig. 9).

Dorsum glabrous. Pronotum (Fig. 14) 1.2–1.3 times wider than long; greatest width slightly anterior of middle. Posterior margin 1.1–1.2 times wider than anterior margin, lateral margin gently convex, posterior margin nearly straight in middle, curved laterally, and narrowly margined. Anterolateral callosity longer than wide, setigerous pore posterolaterally situated, not forming denticle at pore; posterolateral callosity protruding slightly laterally, setigerous pore laterally situated. Disc without impressions, shiny, uniformly punctate, punctures small, smaller than those on elytra. Anterior coxal cavity open behind (Figs 4, 16); intercoxal prosternal process short, acutely pointed, not reaching midlevel of procoxa (Fig. 16); procoxae longer than wide and closely associated; shortest distance from anterior margin of prosternum to procoxal cavity about 1/4–1/5 of longitudinal procoxal diameter. Mesoscutellum triangular, about two times wider than long, flat, impunctate to minutely punctate. Intercoxal mesosternal process short, not reaching midlevel of mesocoxa (Figs 4, 17). Mesepisternum broader than mesepimeron (Fig. 17). Metasternum no longer than first two abdominal ventrites combined (Fig. 4).

Elytra broader than pronotum basally, maximum width posterior of middle. Humeral callus well developed; elytral border narrow, becoming indistinct towards apex; elytral apex broadly rounded; epipleuron (Fig. 4) oblique, maximum width near anterior 1/4 of elytron, maximum width subequal to about 1.5 times maximum width of mid-femur, narrows abruptly before middle and then continues very narrowly, becoming indistinct towards the elytral apex. Hind wings present. Metanotum (Fig. 15) well developed with full complement of internal ridges.

All femora oblong in cross section; all tibiae subcylindrical, subcircular in cross section with a minute apical spur; metatibial spur subequal to claw in length; proportionate length of femur–tibia–tarsomeres 1–4 as follows: 1: 1.0–1.1 : 0.2–0.3 : 0.1–0.2 : 0.1–0.2 : 0.2–0.3 (foreleg); 1: 0.9–1.0 : 0.3 : 0.1–0.3 : 0.1–0.2 : 0.2–0.3 (midleg); 1: 1.1–1.2 : 0.4 : 0.1–0.2 : 0.1 : 0.2 (hindleg); joint where metatibia and first metatarsomere meet, black; third tarsomere always bilobed; claws simple and appendiculate, appendix small and basal. Abdomen (Fig. 4) with five distinct ventrites; ventrites 2–4 becoming progressively slightly shorter; fifth ventrite slightly longer than fourth; intercoxal projection of first abdominal ventrite acute; apical abdominal tergite (Fig. 19, 20) without a median longitudinal groove, posterior margin slightly concave medially in male of *Madurasia undulatovittata* (Fig. 20) and distinctly emarginate in *Madurasia andamanica* sp. n.; posterior margin of apical tergite broadly convex (Fig. 19) in females of both species; posterior margin of apical ventrite more or less lobed medially in male (Figs 22, 23), entire in female.

**Figures 21–27.**
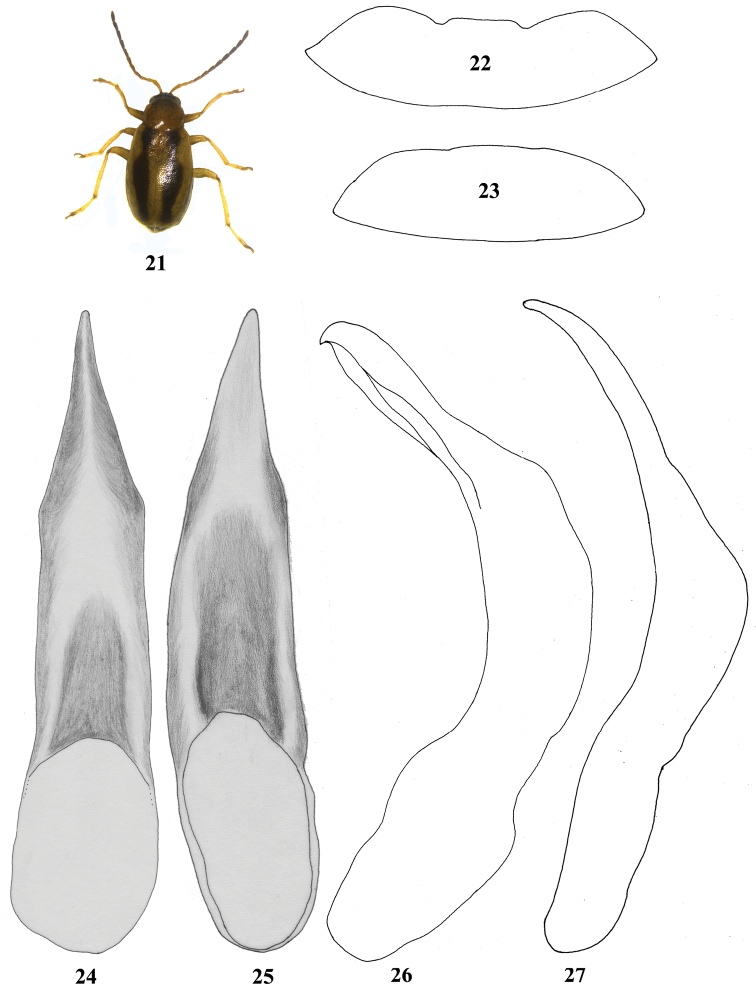
*Madurasia andamanica* sp. n. **21** dorsal habitus **22** apical ventrite of *Madurasia andamanica* sp. n. male **23** apical ventrite of *Madurasia undulatovittata* male **24** median lobe of aedeagus in *Madurasia andamanica* sp. n., ventral view **25** median lobe of aedeagus in *Madurasia undulatovittata*, ventral view (bilaterally symmetrical, specimen tilted) **26** median lobe of aedeagus in *Madurasia andamanica* sp. n., lateral view **27** median lobe of aedeagus in *Madurasia undulatovittata*, lateral view.

**Figures 28–33.**
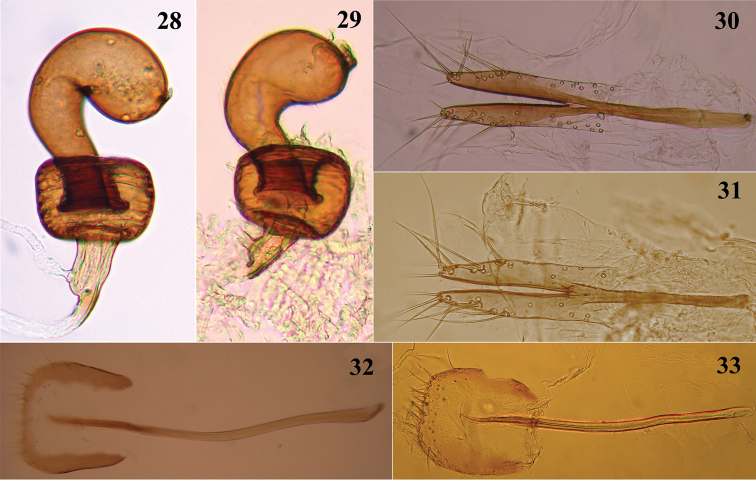
Spermatheca in **28** *Madurasia andamanica* sp. n. **29** *Madurasia undulatovittata*; vaginal palpi of **30** *Madurasia andamanica* sp. n. **31** *Madurasia undulatovittata*; tigna in **32** *Madurasia andamanica* sp. n. **33** *Madurasia undulatovittata*.

Female genitalia with receptacle of spermatheca (Figs 28, 29) pot-shaped, wider than long; pump curved, longer than receptacle and enlarged distally, appendix well developed; spermathecal duct shorter than receptacle, glandular duct beyond middle of spermathecal duct. Tignum (Figs 32, 33) gently curved near middle, grooved medially, with long setae near distal margin of broad membranous apex. Vaginal palpi (Figs 30, 31) fused from proximal end to a short distance beyond middle, separate distally, each palpus narrowing towards rounded apex, lateral margin concave preapically, with long distal setae. Median lobe of aedeagus strongly curved in lateral view (Figs 26, 27), acutely pointed. Tegmen with stem much longer than arms.

#### Host plants.

Fabaceae.

#### Distribution.

Asia, Africa (Sudan).

#### Remarks.

*Madurasia* closely resembles *Medythia* Jacoby, 1887, and species of both genera are pests of legumes. The general morphology, including the structure of the head, female genitalia, and even the presence of elytral stripes in some species of *Medythia*, are similar to those in *Madurasia*, making differentiation of these genera difficult. *Madurasia* can be separated from *Medythia* by the structure of the pronotum. The pronotum in *Medythia* is elongate and narrows posteriorly, whereas the pronotum is transverse and a little wider posteriorly in *Madurasia*. The elytral epipleuron is short in *Madurasia*, hardly extending beyond middle of the elytron. In *Medythia quadrimaculata* Jacoby, type species of the genus, the elytral epipleuron is longer, extending beyond the middle of the elytron. However, the epipleura are identical to those of *Madurasia* in a few Indian *Medythia* species examined. In *Madurasia*, the distal antennomeres are darker, while antennomeres 8–10 are whitish in most *Medythia* species, including the type species.

Adults are attracted to light.

### Madurasia andamanica sp. n.

Taxon classificationAnimaliaColeopteraChrysomelidae

http://zoobank.org/3D810CFF-3113-43FE-8E67-BD8335505E90

Figs 21 , 22 , 24 , 26 , 28 , 30 , 32 , 33

#### Diagnosis.

The new species can be recognized by the following characters: 1) elytral stripe not reaching the elytral apex, narrowing in distal 1/4; 2) labrum with a transverse row of eight well developed setae; 3) posterior margin of apical ventrite in male distinctly lobed medially; 4) apex of aedeagus in lateral view curved like a parrot’s beak with an acute tip; 5) ventral side of aedeagus depressed in basal 1/2, then distally raised in the form of a narrow ridge which reaches the apex.

#### Description.

Body: length 2.1–2.6 mm; width 1.1–1.2 mm; 1.8–2.1 times longer than wide (Fig. 21). Dorsum straw colored. Head dark brown. Antenna with basal three or four antennomeres a pale straw color, distal antennomeres becoming progressively darker. Mandible, maxilla, and labium paler than labrum and anterior aspect. Pronotum with pale orange hue. Elytra a pale straw color, the dark elytral stripe not reaching the elytral apex (Fig. 21), widest anteriad of middle, narrowing distinctly posterior of humerus as well as in distal 1/4. Thoracic sternites and pronotum concolorous, metathoracic sternite often a slightly darker laterally. Abdominal ventrites pale brown, with lateral margins and apical abdominal ventrite darker in many specimens. Legs straw colored, tibia and first two tarsomeres often a slightly darker than femur. Antenna reaching slightly beyond middle of elytron. Proportionate length of antennomeres 1–11: 1: 0.48: 0.45–0.50: 0.63–0.67: 0.63: 0.61–0.62: 0.66–0.67: 0.62–0.70: 0.63–0.69: 0.62–0.63: 0.75–0.88. Transverse diameter of eye 6.3–8.0 times width of orbit, 3.3–3.8 times width of interantennal space, 1.7–1.9 times width of antennal socket, 0.7 times distance between eyes. Pronotum 1.2–1.3 times wider than long, posterior width 1.1–1.2 times wider than anterior width.

Proportionate length of femur:tibia:tarsomeres 1–4 as follows: 1: 1.0–1.1 : 0.2–0.3 : 0.2 : 0.1–0.2 : 0.2–0.3 (foreleg); 1: 0.9–1.0 : 0.3 : 0.1–0.3 : 0.1–0.2 : 0.2–0.3 (midleg); 1: 1.0–1.1 : 0.4 : 0.1–0.2 : 0.1 : 0.2 (hindleg).

Posterior margin of apical ventrite in male distinctly lobed medially (Fig. 22). Receptacle of spermatheca 2.4 times wider than long (Fig. 28). Tignum widened proximally; membranous apex wider towards posterior (Fig. 32).

Aedeagus in lateral view (Fig. 26) with greatest width near middle, narrow in proximal 1/4, apex curved like a parrot’s beak with an acute tip. In ventral view (Fig. 24), greatest width at base, narrowing abruptly in apical 1/3; ventral aspect depressed in basal 1/2, then distinctly raised in the form of a narrow ridge which reaches the apex.

#### Etymology.

Named after the Andaman Islands, where the new species occurs.

#### Material examined.

**Holotype** ♂ “INDIA: Andaman & Nicobar / North Andaman: Diglipur / 13°14'53.9"N, 92°58'37.5"E, / 15 mts. 24.iv.2014. At light / Yeshwanth H. M.” (white label); “HOLOTYPE / Madurasia andamanica / Prathapan sp. nov., 2015” (red label) (BMNH).

Paratypes (104). 5♂, 8♀ same data as holotype; 7♂, 19♀ same data as holotype, but 23.iv.2014; 2♀ INDIA: Andaman & Nicobar / South Andaman: Sippighat / 11°67'26"N, 92°67'12"E, / 44 mts. 18.iv.2014, Light trap / Yeshwanth H. M.; 1♂, 16♀ India: South Andaman / Garacharama / 12.xi.2014 / Bharathimeena Coll. / Ex Redgram; 16♂, 7♀, 1 unsexed same data but 8.I.2015; 2♂, 20♀ same data but 4.XII.2014 and collector Krishnaveni (5 BMNH, 5 USNM, 5 JBC, 5 KAU, 5 UASB, 40 NBAIR, 39 INPC).

#### Distribution.

**Figure 34.**
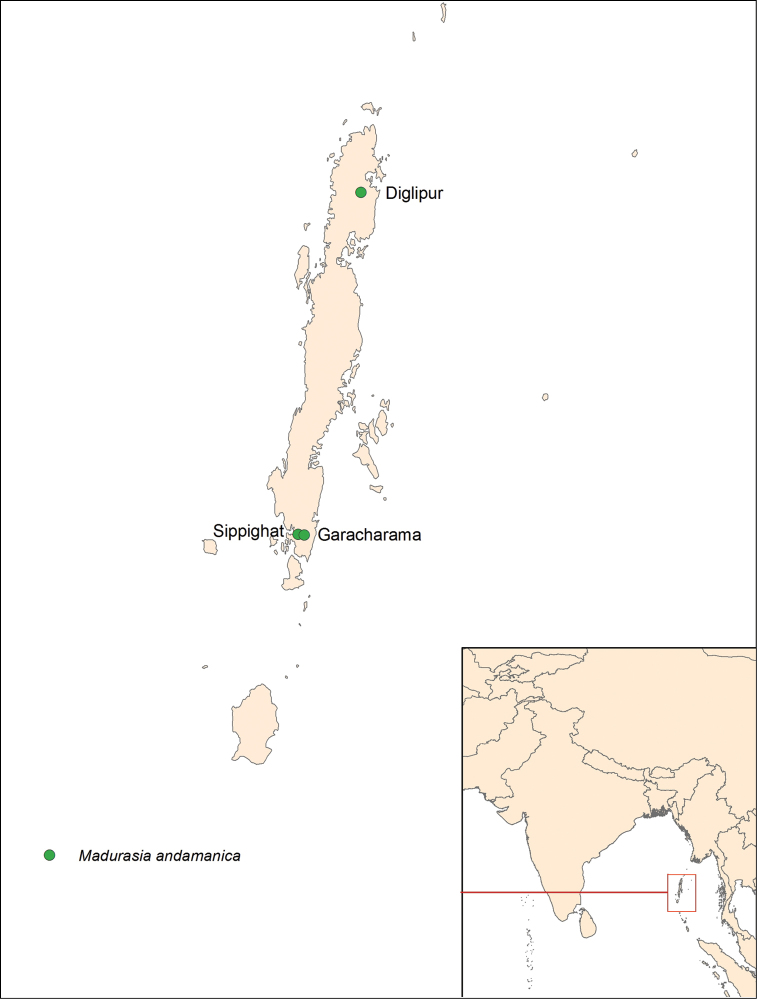
Distribution of *Madurasia andamanica* sp. n. on the Andaman Islands.

India (Andaman Islands) (Fig. 34).

#### Remarks.

Color pattern in *Madurasia andamanica* sp. n. (Fig. 21) appears to be consistent and less variable compared to that in *Madurasia undulatovittata* (Motschulsky, 1866), where the color of specimens collected on the same host at the same locality on the same day varied greatly. *Madurasia andamanica* sp. n. resembles *Madurasia undulatovittata* externally. However, it can be distinguished based on the structure of the aedeagus and the number of labral setae, as described under *Madurasia undulatovittata*.

#### Host.

*Cajanus cajan* (L.) Millsp. (Fabaceae) (red gram or pigeon pea) (Bharathimeena T., pers. comm. 2015).

### Madurasia undulatovittata

Taxon classificationAnimaliaColeopteraChrysomelidae

(Motschulsky) comb. n.

Figs 1 , 3–7 , 8–20 , 23 , 25 , 27 , 29 , 31 , 33 , 35

Teinodactyla undulatovittata Motschulsky, 1866: 417 [Sri Lanka, Lectotype (ZMUM)]–Wagner and Bieneck 2012: 214–215.
Longitarsus undulatovittatus : Gemminger and Harold 1876: 3509–Maulik 1926: 361.
Monolepta undulattovittata : Ogloblin 1930: 112.
Madurasia obscurella Jacoby, 1886: 381 [“Madura, Madras Presidency”, Southern India– Lectotype (BMNH)]–Maulik 1936: 74–Wilcox 1973: 435–Takizawa 1987: 39–Takizawa and Kimoto 1990: 8–Takizawa 1990: 281–Mohamedsaid 1997: 5–Medvedev and Sprecher 1999: 310 (catalogue)–Mohamedsaid 2000: 370–Kimoto 2005: 58–Beenen 2010: 481–Bezděk 2012: 422, 424. **New synonym.**
Neorudolphia bedfordi Laboissière, 1926: 191 [Brit. Sudan, on Cajanus indicus, Syntype (ZMUH, Hamburg)]–Aslam 1972: 500 (synonymized with Madurasia obscurella Jacoby)–Wilcox 1973: 435–Weidner 1976: 229.

#### Description.

Body: length 2.0–3.0 mm; width 1.0–1.3 mm; 2.0–2.3 times longer than wide. General color pattern consistent but highly variable in intensity (Figs 1, 3, 5–7). Head dark brown to pale brown, often darker than pronotum. Basal antennomeres 3–6 pale straw brown, distal antennomeres becoming progressively darker. Pronotum more or less pale brown, generally paler than head. Background color of elytron paler than pronotum. Lateral margin of dark elytral stripe emarginate in anterior 1/3 and posterior 1/3; stripe broadening posteriorly, covering width of elytral apex. In some specimens, elytra darker laterally giving the impression of a pale, medially narrowed line on a dark elytron. Intensity of stripe’s darkness varies from pale straw brown (Fig. 1) to dark brown. In type of *Madurasia undulatovittata*, elytral stripes are hardly visible (Fig. 1). In some specimens, widest region in middle of stripe extends to lateral elytral margin, thus dividing pale colored lateral area into anterior and posterior spots (Figs 6, 7). Ventral aspect (Fig. 4) generally paler than head. Metasternum slightly darker than pro– or mesosternum. Metepisternum darker than metasternum. Abdomen darker laterally and posteriorly in many specimens. In darkest specimens, ventral side dark brown to piceous. Legs pale brown, all femora nearly concolorous with abdominal ventrites; metafemora darker distally in some specimens. All tibiae paler than femora. Metatibia and first metatarsomere whitish in some specimens. Claw tarsomere and bilobed tarsomere often darker than preceding ones.

Antenna (Fig. 9) reaches middle of elytron or a little beyond. Proportionate length of antennomeres 1–11: 1: 0.54–0.57 : 0.44–52 : 0.65–0.69 : 0.59: 0.66: 0.66–0.69: 0.62–0.75: 0.73–0.75: 0.69–0.72: 0.69–0.71: 0.81–0.91. Transverse diameter of eye 5.3–8.8 times width of orbit, 2.9–4.4 times width of interantennal space, 1.7–1.8 times width of antennal socket, 0.6 times distance between eyes. Pronotum (Fig. 14) 1.3 times wider than long, posterior 1.1 times wider than anterior.

Proportionate length of femur–tibia–tarsomeres 1–4 as follows: 1: 1.0–1.1 : 0.3 : 0.1–0.2 : 0.1–0.2 : 0.3 (foreleg); 1: 0.9–1.0 : 0.3 : 0.1–0.2 : 0.1–0.2 : 0.2–0.3 (midleg); 1: 1.1–1.2 : 0.4 : 0.1–0.2 : 0.1 : 0.2 (hindleg). Two visible apical tergites completely exposed in most females, particularly when killed in alcohol.

Posterior margin of apical ventrite in male (Fig. 23) indistinctly lobed medially. Receptacle of spermatheca 1.6 times wider than long (Fig. 29). Tignum not widened proximally (Fig. 33); membranous distal region widest medially.

Aedeagus in lateral view (Fig. 27) strongly curved after basal 1/2, acutely narrowed in proximal 1/3, with weakly curved apex. In ventral view (Fig. 25), widest in proximal 1/3, narrowing sharply towards apex in apical 1/3, lateral margin a little abruptly narrowed preapically. Ventral aspect of aedeagus depressed with a convex portion in middle.

#### Material examined.

**Types.** *Madurasia undulatovittata*: Lectotype ♀. “Teinodactila / undulato / vittata Motch / Ceylon”; “Monolepta / undulatovittata Mots. / 1926 D. Ogloblin det.”; “LECTOTYPUS / des Döberl 2005” (ZMUM).

*Madurasia obscurella*: Lectotype ♀. “Type” (rectangular red label); “Andrewes / Bequest. / B. M. 1922–221.”; “Madura”,“738” (3 in 738 is not legible as pierced by pin); “Madurasia obscurella Jac. /Type”; “SYNTYPE” (white circular disc with sky blue margin); “Lectotype / Madurasia obscurella Jacoby / des. K. D. Prathapan, 2015” (here designated, specimen on card, right antenna missing) (BMNH).

Paralectotype ♀. “Type / H. T.” (white circular disc with red border); “Madura”; “Jacoby Coll. 1909–28a.”; “Madurasia / obscurella / Jac. Type” (Blue label); “SYNTYPE” (white circular disc with sky blue margin); “Paralectotype / Madurasia obscurella Jacoby / des. K. D. Prathapan, 2015” (BMNH).

#### Non-type material.

**AFRICA: Sudan**: ♀ British Sudan, S. R. J. Madani, 22.ix.1923, H. M. Bedford, feeding on ‘adis’ (illegible) sudani leaves / Blue Nile A 3024 / Pres by Imp. Bur. Ent. Brit. Mus. 1925–228 / standing as Neorudolfia (sic) bedfordi; 1 unsexed R. F. Wadmedanai J. W. Cowland 21/9/32 Shotholing seedlings of Phaseolus mungo / Ent. Coll. C 12147 / AFRICA 250,000 55–G Map / Pres. by Imp. Inst. Ent. BM 1933–415 / Standing as Neorudolphia bedfordi / SUDAN Govt.; 1 unsexed Blue Nile 5429 / Aenk H. H. & D. King 26.5.13 On boot / Pres. by Imp. Bur. Ent. Brit. Mus. 1927–103 / Neorudolphia bedfordi V. Laboissière–Dèt. (all BMNH).

**ASIA: Bangladesh**: ♀ (India) Dacca, 2.vi.1945, D. Leston; ♀ (India) Dacca, 10.v.1945, D. Leston (both BMNH); **India**: *Andhra Pradesh*: 3 unsexed Vizagapatnam Dist., Chipurupalli, B.M. 1924–7; *Gujarat*: 2♀ Baruch, 10.xii.1987, Pigeon pea, CIE A19617; Navasari, 15.iii.1992, Assoc with cowpea, IIE 22432, Madurasia obscurella Jac det. M. L. Cox 1992 (all BMNH); *Karnataka*: 1 macerated specimen Belgaum, 1–2.viii. 2008, at light, K. Swamy; 2♀, 1♂ Chikkaballapur, 13°25'48"N, 7°43'12"E 694 mt., 29.viii.2010, Nirmala P., at light (all UASB); *Kerala*: 7♂, 2 ♀ Vellayani, N 08°25'47.5"E, 76°59'8.3", 21.vii.2015, 18 m, Prathapan KD; 19 ♀ , same data except for the date 5.vii. 2015 and ex Green gram (NBAIR, JBC, INPC, BMNH); *Maharashtra*: 3♀ Bandra, Jayakumar, 1905–152; 2 unsexed Bombay (Mumbai), 79.15 ; 1♀ Bombay, G. Bryant, 1919–147; 1 unsexed, Poona (Pune), 27.viii.1944, D. Leston, BM 1946–365; 1♀ 21.x.1944, D. Leston, BM 1945–86 (all BMNH); *Meghalaya*: 1♂, 1 ♀ SW of Cherapunjee, 23°13'15"N/ 91°40'E, 500–900 m, 11–12.v.2004, R. Businsky (all JBC); *New Delhi*: 4♀, 6 unsexed 21.viii.1968, on cowpea, Phaseolus and urd (all BMNH); *Rajasthan*: 1♀Jodhpur N 26°21'4.6"E, 73°2'39"5.VIII.2015 255 m, Prathapan K. D. (KAU); 10 unsexed Banswara 24.ix.2015, S. Ramesh Babu (KAU); *Uttar Pradesh*: Saharanpur Div., Siwalik Hills, 8.iv.1928, H. G. Champion, B.M. 1928–518 (BMNH); *Uttarakhand*: 1 ♀ Dehra Dun, 8.ix.’16, H. G.Champion, BM. 1953– 156; 1 ♀ Ranikhet, 6–8. ’16, H. G.Champion, BM. 1953–156 (both BMNH); *West Bengal*: 2 unsexed Sarda, H. G. Champion, B.M. 1953–156; 2 unsexed Sunderbans, H. G. Champion, B.M. 1953–156 (all BMNH); **Sri Lanka**: 2 unsexed, 1♀ Girandurukotte no. 68, 16.xii.86 on cowpea, CIE A18795; 2♀ Maha Illupallama, 1976, R. W. Fellowes, R. W. Fellowes, on Glycine & Vigna, CIE A9047 (all BMNH); **Yemen**: 2 ♀ Al Hudaydah gov., Jabal Bura Valley forest N. P., (stream valley), 240–350 m, 15°52.4–5'N, 43°24.6–25.2'E, J. Bezdӗk, 4.xi.2010; 1♂, 2♀ Socotra Island, wadi Ayhaft, 12°36.5'N, 53°58.9'E, 200 m, J. Bezdӗk, 7–8.xi.2010 (all JBC).

#### Distribution.

**Figure 35.**
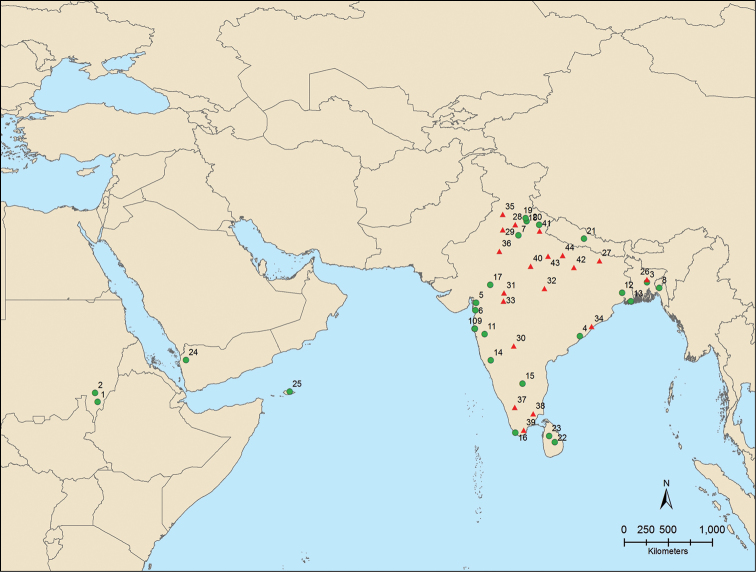
Distribution of *Madurasia undulatovittata* (Motschulsky) in the Afrotropical and Oriental regions (red triangles = literature records).

Africa (Sudan); Asia (Bangladesh, India [Andhra Pradesh, Bihar, Gujarat, Haryana, Karnataka, Kerala, Madhya Pradesh, Maharashtra, Meghalaya, New Delhi, Orissa, Punjab, Rajasthan, Tamil Nadu, Uttar Pradesh, Uttarakhand, West Bengal], Nepal, Sri Lanka, Yemen) (Fig. 35).

#### Remarks.

*Madurasia undulatovittata* and *Madurasia andamanica* sp. n. are very similar. However, they can be separated as follows: eight labral setae present in *Madurasia andamanica* sp. n. (only six labral setae visible in *Madurasia undulatovittata*, though eight pores are present); elytral stripes are highly variable in *Madurasia undulatovittata*, even in specimens from the same locality, collected during the same season and on the same host. The elytral pattern in *Madurasia andamanica* sp. n. is rather consistent. The stripe in *Madurasia undulatovittata* is wider apically in specimens where it is well defined, while in *Madurasia andamanica* sp. n., it is narrowed apically. In *Madurasia andamanica* sp. n., the stripe is distinct and well defined against the pale background color. Verma (1995) recorded variation in elytral color pattern. Lobe in the middle of the posterior margin of the apical abdominal ventrite in males distinct in *Madurasia andamanica* sp. n., but poorly distinguishable in *Madurasia undulatovittata*. The two species can easily be separated by the structure of the aedeagus. In lateral view, the apex of aedeagus of *Madurasia andamanica* sp. n. is curved and pointed, like the beak of a parrot (Fig. 26), while the same in *Madurasia undulatovittata* is narrowly rounded, and smoothly curved in apical 1/3 (Fig. 27). The sharply raised ridge on the ventral aspect of the aedeagus in *Madurasia andamanica* sp. n. (Fig. 24) is characteristic, however, this ridge is absent in *Madurasia undulatovittata* (Fig. 25). *Madurasia andamanica* sp. n. is confined to the Andaman Islands and reported to feed on pigeon pea, while *Madurasia undulatovittata* is transcontinental in distribution and a significant pest of a number of species of pulses in southern Asia and Africa (Sudan).

A photograph of the labels provided by Wagner & Bieneck (Fig. 38a in Wagner and Bieneck 2012) shows three labels, two of which show different information for *Madurasia undulatovittata* (Fig. 2). Labels currently on the specimen indicate that M. Döberl designated the lectotype in 2005. However, no publication by Döberl could be traced in which this specimen is mentioned. According to Wagner and Bieneck (2012), the lectotype was designated by Wagner, and they provide photographs of both the lectotype and its labels. The photograph (Fig. 38b) in Wagner and Bieneck (2012), confirms that the specimen examined by me is the one designated as lectotype by Wagner (Fig. 1). Moreover, Wagner and Bieneck (2012) also mention that the only other specimen, a paralectotype in Motschulsky’s collection, is a male from which the aedeagus has been dissected and subsequently lost. Dr Wagner’s lectotype designation stands valid as that alone is published (Wagner and Bieneck 2012). Dr Döberl designated the same specimen as lectotype in 2005 as there was a long gap of nearly a decade between the lectotype designation by Dr Wagner and the publication of the same in Wagner and Bieneck 2012 (T. Wagner and M. Döberl, pers. comm., 2016). The specimen collected by Bedford on 22.ix.1923, identified as *Neorudolphia bedfordi* by Laboissière from the BMNH, probably belongs to the type series of *Neorudolphia bedfordi*. The lectotype for *Madurasia obscurella* is here designated, to have a unique name bearer and standard for its application.

#### Host plants.

Fabaceae: *Cajanus cajan* (L.) Millsp. (red gram or pigeon pea); *Glycine max* (L.) Merr. (soybean); *Lablab purpureus* (L.) Sweet (= *Dolichos lablab* L.) (lablab bean); *Vigna aconitifolia* (Jacq.) Marechal (moth bean); *Vigna mungo* (L.) Hepper (= *Phaseolus mungo* L. = *Phaseolus radiatus* Roxb. non L.) (black gram); *Vigna radiata* (L.) R. Wilczek (= *Phaseolus aureus* Roxb. = *Phaseolus radiatus* L.) (green gram or moong); Vigna radiata (L.) Wilczek var. sublobata (Roxb.) (= *Phaseolus sublobatus* Roxb.); *Vigna umbellata* (Thunb.) Ohwi & Ohashi (rice bean) and *Vigna unguiculata* (L.) Walp. (= *Vigna sinensis* (L.) Savi ex Hausskn.) (cowpea).

#### Biology and management.

Information on the host plants and biology of *Madurasia undulatovittata* was generated by agricultural entomologists in India, under the name *Madurasia obscurella*, where it is a widely distributed pest of legume crops across many agro climatic zones. The first record of this species as a pest of pulses is that by Menon and Saxena (1970). According to Naresh and Thakkur (1972), it was reported as a major pest of black gram by Naresh and Nene in 1968. However, there is no mention of this leaf beetle in Naresh and Nene (1968). Saxena et al. (1971) described it as a pest of cowpea, green gram or moong and black gram or urd, indicating that it made holes in the leaf lamina. Other recorded host plants include *Glycine* (CAB International Institute of Entomology 1990), moth bean (Pareek et al. 1983), lablab bean (Gupta and Singh 1984a, b), pigeon pea (Saxena 1977, Mishra and Saxena 1983), rice bean (Satyanarayana et al. 1995a, b) and Vigna radiata (L.) Wilczek var. sublobata (Roxb.) (=*Phaseolus sublobatus* Roxb.) (Kalaichelvan and Verma 2005).

Gupta and Singh (1984a, b) provided the first account of its life cycle. They recorded the total life cycle as varying between 32 and 44 days and that it completes two generations a year on green gram. A second, more detailed study of the life history was reported by Oza et al. (1996) on cowpea. Eggs were laid singly on soil near the root zone of the plant. The total duration of the life cycle, from egg to death of adult, varied between 35 and 48 days in males and 43 to 58 days in females.

The growth of plants is retarded by severe foliage injury, especially in young plants (Srivastava and Singh 1976). Leaf damage on green gram in summer and rainy season crops ranged between 5–10% and 15–50% respectively (Sinha et al. 1985). Larvae are soil dwelling and feed on root hairs (Srivastava and Singh 1976; Gupta and Singh 1981). Odak and Thakur (1978) reported larval feeding on the root nodules. Gowda and Kaul (1982) recorded adult feeding on leaves, buds and flowers. Gowda et al. (2006) also observed feeding damage by adults on the buds and flowers of pigeon pea. Reddy and Varma (1986) established transmission of southern bean mosaic virus in cowpea by *Madurasia undulatovittata*. The success in transmission varied from 25 to 43%.

The extent of damage on black gram, green gram and cowpea was 20–60% (Srivastava and Singh 1976). This is a common pest of mung bean in the first crop season (*kharif*) in India, coinciding with the southwest monsoon (June to October) (Tiwari 1978). Singh and Gupta (1982) estimated damage to the leaves of green gram and black gram. Infestation was more pronounced in black gram than in green gram. Infestation starts when the plants are in the two leaf-stage and the insects remain active until flowering (Dhuri and Singh 1983, Nayak et al. 2005).

In Haryana, Yadav and Yadav (1983) recorded it from cowpea and Mrig and Singh (1985) observed maximum damage on *Dolichos lablab* during the third week of September, with the pest disappearing after the first week of November. Feleiro and Singh (1985) carried out yield–infestation studies to fix the critical stages of crops requiring protection. They observed that infestation in summer resulted in heavy yield losses, while the pest attack during the rainy season had no significant effect on yield.

Lal (1985) reviewed information on the biology and control of insect pests of mung bean, including *Madurasia undulatovittata*, in India.

According to Faleiro et al. (1986), *Madurasia undulatovittata* is a sporadic, but major pest of cowpea. A peak population of 10.0–10.25 beetles/10 plants in summer and 29.50–30.25 beetles/10 plants in the rainy season were recorded by Gupta and Singh (1993) in green gram. Sahoo and Patnaik (1994) recorded the incidence of insect pests in green and black gram, and their seasonal activity and the extent of damage in Orissa. *Madurasia undulatovittata* was severe on both the crops in the seedling and vegetative stages, and was the first pest to appear at seedling stage on rice bean, continuing to occur until flowering (Satyanarayana et al. 1995b). Ganapathy and Durairaj (1995) reported it as an important pest on black gram and green gram in drought prone Pudukottai District, Tamil Nadu. There was more damage in black gram (9.78%) than in green gram (1.45%). However, there are also reports of *Madurasia undulatovittata* only being a minor pest (Devesthali and Joshi 1994, Kumar et al. 1998).

Dhuri et al. (1984) observed population buildup of *Madurasia undulatovittata* under ambient temperature of about 32°C, longer duration of bright sunshine and high relative humidity coupled with intermittent rainfall. Sardana and Verma (1986) showed that maximum temperature and sunshine were negatively, but significantly, correlated with the population of the pest, while rainfall showed a significantly positive correlation. Maximum temperature, minimum temperature, sunshine hours and wind velocity had a significantly negative correlation with damage (Irulandi and Balasubramanian 1999). Nayak et al. (2004) reported a significantly negative correlation with minimum temperature and relative humidity during population buildup on black gram. The population did not show any correlation with maximum temperature, relative humidity and rainfall, but it was highly and significantly correlated with minimum temperature (Kumar et al. 2007).

Various cultivars of green gram (Srivastava et al. 1975, Sahoo et al. 1989, Sahoo and Hota 1991) and black gram (Sahoo et al. 1989) vary significantly in their susceptibility to the pest. Pandey et al. (1995) reported that varieties with thicker leaves were preferred by the pest.

Chemical control remains the most effective option against this pest. Several broad spectrum insecticides have been tried against *Madurasia undulatovittata*, with varying degrees of success (Saxena et al. 1971, Naresh and Thakur 1972, Saxena et al. 1975, Verma and Pant 1975, Verma and Lal 1976, Vyas and Saxena 1977, Verma and Lal 1978, Yadav et al. 1979, Vyas and Saxena 1981, Chaudhary et al. 1981, Rajendran et al. 1981, Vyas and Saxena 1982, Mishra and Saxena 1983, Singh et al. 1983, Singh and Gupta 1984, Sinha 1985, Gattoria and Singh 1988, Rahman 1988, 1991a, b, Chander and Singh 1989, Sinha and Sharma 1989, Verma and Dikshit 1990, Logiswaran and Gopalan 1993, Uddin et al. 1994, Das 1999, Gowda et al. 2006 and Pandey et al. 2007). Application of neem seed kernel extract had no significant effect to increase yield in mung bean (Yadav et al. 1979). Soundararajan and Chitra (2011) tried biological control agents such as *Pseudomonas flourescens* and *Beauveria bassiana* and reported that intercropping black gram with sorghum reduced infestation (Soundararajan and Chitra 2012).

## Discussion

A revision of the genus *Medythia* is required to define the boundaries between it and the genus *Madurasia*. *Medythia* and *Madurasia* share the same ecological niche and are often collected together on the same host plants, as well as at light. It is likely that economic entomologists have often misidentified one for the other. *Medythia bukit* and *Medythia marginicollis*, described by Mohamedsaid (1999) from Malaysia, with pronotum broader than wide, as well as a little narrower anteriorly than posteriorly, appear atypical for the genus. Reports of a 6.05mm, ovate beetle as *Madurasia obscurella* from Pakistan (Rizvi et al. 2012, Kamaluddin et al. 2012) are incorrect.

*Madurasia andamanica* sp. n. is a significant pest of red gram or pigeon pea (*Cajanus cajan*) in the Great Andaman Islands (Bharathimeena T., pers. comm. 2015), similar to the pest status of *Madurasia undulatovittata* elsewhere. This endemic pest of the islands, in case of accidental introduction to the mainland India, is likely to become a pest of various pulses and spread far and wide, as in the case of *Madurasia undulatovittata*.

## Supplementary Material

XML Treatment for Madurasia

XML Treatment for Madurasia andamanica

XML Treatment for Madurasia undulatovittata
